# Clinical effect of kyphoplasty in the treatment of osteoporotic thoracolumbar compression fractures in patients with diabetes

**DOI:** 10.3389/fsurg.2022.1031547

**Published:** 2023-02-07

**Authors:** Xiangcheng Gao, Jinpeng Du, Yunfei Huang, Shuai Li, Dingjun Hao, Baorong He, Liang Yan

**Affiliations:** ^1^ Medical College, Yan'an University, Yan'an, China; ^2^ Department of Spine Surgery, Honghui Hospital, Xi'an Jiaotong University, Xi'an, China

**Keywords:** kyphoplasty, diabetes, thoracolumbar fracture, clinical effect, risk facors

## Abstract

**Objective:**

To study the clinical effect and influencing factors of kyphoplasty in the treatment of osteoporotic thoracolumbar compression fractures (OTCF) complicated with type 2 diabetes mellitus (T2DM).

**Methods:**

A total of 472 patients with OTCF complicated with diabetes who were enrolled in our hospital from January to December 2019 were selected as the study subjects, and all patients were treated with percutaneous kyphoplasty (PKP). The effects of gender, age, smoking, drinking, body mass index (BMI), bone mass density (T score), fasting blood glucose level, fasting C-peptide, glycosylated hemoglobin, course of T2DM, vertebral segment and surgical instrument on postoperative improvement were analyzed. The quality of life was evaluated by visual analog score (VAS) and Oswestry disability index (ODI) before PKP and 7 days, and 6 months after PKP, and the patient satisfaction was assessed by the modified Macnab criteria at 6 months postoperatively.

**Results:**

The overall excellent and good rate of evaluation result was satisfactory. In multivariate regression, independent risk factors for poor patient satisfaction included: age ≥70 years (odds ratio (OR) = 2.298, 95% confidence interval [CI] 1.290–4.245, *P* = 0.025), fasting blood glucose ≥8 mmol/L [OR = 2.657, 95%(CI) 1.288–4.121, *P* = 0.016], glycosylated hemoglobin ≥6.5 mmol/L [OR = 3.438, 95%(CI) 2.543–4.628, *P* = 0.001], duration ≥8 years [OR = 1.732, 95%(CI) 1.471–3.253, *P* = 0.019] and Kyphon instrument [OR = 1.472, 95%(CI) 1.112–2.228, *P* = 0.018] were independent influencing factors of OTCF complicated with DM.

**Conclusion:**

Kyphoplasty for patients with osteoporotic thoracolumbar compression fractures complicated with diabetes can achieve a satisfactory clinical effect, the curative effect is affected by many factors, attention to these factors can improve the clinical effect.

## Introduction

Diabetes mellitus (DM) and osteoporosis are major public health problems in the aging population affecting people all over the world and are of serious health and financial concern (1). DM can cause great harm to the human body, among which chronic complications of skeletal system have a high rate of death and disability, which can cause long-term bone pain and lead to diabetic osteoporosis (2). Diabetic osteoporosis is characterized by both diabetes and osteoporosis, and generally occurs in elderly patients with a long history of diabetes. The fracture sites are mostly thoracolumbar vertebrae and hip, and currently percutaneous kyphoplasty (PKP) is the most common method for the treatment of osteoporotic thoracolumbar compression fractures (OTCF) (3). This is a method of percutaneous injection of bone cement into the vertebral body through the pedicle to increase the strength and stability of the vertebral body. It can effectively prevent and cure the collapse of the vertebral body and greatly relieve the pain of the patient. Although previous studies have shown that perioperative hyperglycemia, especially diabetes mellitus, is an important marker of adverse events in patients undergoing surgery (4), to our knowledge, the clinical effect of kyphoplasty for OTCF combined with diabetes has not been investigated till now. Therefore, the present study investigated the clinical efficacy of PKP surgery in diabetic with osteoporotic thoracolumbar compression fractures complicated, and further analyzed the influencing factors.

## Materials and methods

### General information

This was a retrospective cohort study, which included 472 patients with OTCF complicated with diabetes at our hospital from January to December 2019. We reviewed each patient's clinical records and built the database.

Inclusion criteria: (1) OTCF was confirmed by low-intensity signal changes on T1-weighted image, high-intensity changes on T2-weighted image preoperatively; (2) bone mass density (BMD) T score≤−2.5 SD; (3) OTCF patients who underwent PKP surgery; (4) patients with T2DM; (5) follow-up time >6 months; (6) complete clinical and imaging data.

Exclusion criteria: (1) acute vertebral compression fracture caused by severe trauma such as car accidents and falling injury; (2) primary or metastatic tumors; (3) multiple myeloma or other systemic diseases; (4) infection; (5) previous spinal surgery; (6) allergic to surgical supplies. This study was approved by the Ethics Committee of Xi'an Honghui Hospital, and all patients obtained preoperative informed consent.

### Surgical methods

All patients were placed in prone position on a spinal reduction stent prior to surgery to maximize reduction of the compressed vertebra. Routine skin disinfection was carried out in the surgical area, sterile towel sheets were laid down and skin care membranes were pasted. The compressed vertebral body was fluoroscopically located, local anesthesia was performed with 1% lidocaine, and incisions about 5 mm long were made on either side of the injured vertebral body. Under fluoroscopy, the pedicle surface of the injured vertebral body was slotted using the apex, and the apex was driven into the pedicle. Pull out the inner core of the apex and insert the guide wire into the vertebral body under fluoroscopy. At this point, pull out the sleeve of the apex vertebra and insert it into the channel along the direction of the guide needle under fluoroscopy. Pull out the guide pin and insert the drill bit into the channel to drill. After that, the drill bit was taken and put into the dilator, and the contrast agent was injected into the balloon. Under fluoroscopy, whether the contrast agent leaked into the blood vessel was observed. After seeing the balloon, it was opened to make the fracture part reset. Remove the contrast agent from the balloon and draw out the dilator. The bone cement sleeve was inserted into the channel, and an appropriate amount of bone cement was injected into the vertebra from both sides of the pedicle under fluoroscopy, and satisfactory filling effect was achieved. The sleeve and channel were pulled out after the cement hardened. The incision was sutured and covered with compresses. All patients were prescribed calcium tablets (600–1,200 mg/day), calcitriol (0.25–0.5 μg/day) and alendronate (70 mg/week), postoperatively.

### Observation indicators

The effects of gender, age, smoking, drinking, BMI, BMD T score, fasting blood glucose level, fasting C-peptide, glycosylated hemoglobin, course of T2DM, vertebral segment and surgical instrumentation postoperative improvement were analyzed. The Visual Analog Scale (VAS) and the Oswestry Disability Index (ODI) were routinely administered to all patients. Meanwhile, the quality of life was evaluated by VAS scores for low back pain and ODI indices for the functional assessment preoperatively, 7 days and 6 months postoperatively, and the modified Macnab criteria were used to assess the patient satisfaction (5). According to the patient satisfaction results on 1 week postoperatively, the patients were divided into the satisfied group (excellent and good) and the dissatisfied group (pair and poor) by the modified Macnab criteria, and the influencing factors were analyzed by multivariate logistic regression.

### Statistical methods

EpiData3.5 software (Epidata Association, Odense, Denmark) was used to establish the database. All analyses were conducted using SPSS version 26 (IBM, Armonk, NY, United States) and GraphPad Prism version 9.3.0 (San Diego, CA, United States). Specifically, Student *t*-test, Mann–Whitney *U*-test, chi-square test, and Fisher exact test were used to evaluate differences between these groups. Multivariate binary Logistic regression analysis were performed under single factor. *P*-value < 0.05 was considered statistically significant.

## Results

### Demographic data

In this study, a total of 472 patients meeting the criteria were included with a follow-up period of 6 months. The general characteristics of the patients are summarized in Table 1. There were 134 males and 338 females in the study, aged from 60 to 85 years old, with an average of 70.5 ± 8.6 years old. The mean BMI and BMD were 22.4 ± 3.14 kg/m^2^ and −3.0 ± 0.3 SD, respectively. The mean fasting blood glucose, fasting C-peptide and glycosylated hemoglobin were 8.0 ± 2.4 mmol/L, 0.67 ± 0.24 nmol/L and 6.5 ± 1.3 mmol/L. The mean course of disease was 8 ± 1.5 years. In terms of instrumentation, KMC system was used in 334 patients and Kyphon was used in 138 patients. There were 108 cases with T11 fracture, 120 cases with T12 fracture, 138 cases with L1 fracture, 106 cases with L2 fracture.

**Table 1:** Descriptive statistics of all patients (*n* = 472).

Characteristics	
Gender (male/female)	134/338
Age (years)	70.5 ± 8.6
Smoking (yes/no)	160/312
Drinking (yes/no)	94/378
BMI (kg/m^2^)	22.4 ± 3.14
BMD (T score)	−3.0 ± 0.3
Fasting blood glucose (mmol/L)	8.0 ± 2.4
Fasting C-peptide (nmol/L)	0.67 ± 0.24
Glycosylated hemoglobin (mmol/L)	6.5 ± 1.3
Duration (years)	8 ± 1.5
Instrument (KMC/Kyphon)	334/138
Fracture level (T11/T12/L1/L2)	108/120/138/106

### Follow-up results

All patients were followed up for 6 months. Preoperative and postoperative VAS scores of low back pain and ODI evaluations are shown in Table 2. The postoperative VAS and ODI scores decreased significantly for all patients. The VAS score of low back pain decreased from the preoperative values of 8.6 ± 0.7 points to 2.3 ± 0.5 points at 6 months postoperatively with a mean decrease of 6.3 ± 0.2 (*P* < 0.001). The ODI score was 72.5 ± 17.7 preoperatively, which declined to 30.2 ± 16.8 at the final follow-up with a mean decrease of 42.3 ± 9.8 (*P* < 0.05). According to the modified Macnab criteria for patient satisfaction, the clinical good-to-excellent rate was 93.6% for all parentis: excellent- 228 (48.3%), good- 214 (45.3%), fair- 20 (4.2%), and poor- 10 (2.1%). Among the follow-up cases, there were 26 cases of adjacent vertebral body fracture and 4 cases of distal vertebral body fracture.

**Table 2:** Comparison of clinical indicators before and after surgery.

Factors	Case	VAS	ODI
Postoperatively (1)	472	8.6 ± 0.7	72.5 ± 17.7
7 days postoperatively (2)	472	2.1 ± 0.6	29.5 ± 15.6
6 months postoperatively (3)	472	2.3 ± 0.5	30.2 ± 16.8
*F* value		238.82	243.96
*P* value		<0.001	<0.001
Paired comparison	* *	*P* value	*P* value
(1) : (2)		<0.001	<0.001
(1) : (3)		<0.001	<0.001

### Results of factors influencing postoperative clinical effect

The results of univariate analysis were shown in Table 3, there was no significant difference in gender, smoking, drinking, BMI, BMD and fracture level between satisfied group and dissatisfied group (*P* > 0.05). Age, fasting blood glucose, fasting C-peptide, glycosylated hemoglobin, duration and instrument were correlated with patient satisfaction after PKP (*P* < 0.05). As shown in Table 4, multiple Logistic regression analysis result showed that the independent influencing factors that positively correlated with patient satisfaction on six month were as follows: age [OR = 2.298, 95%(CI) 1.290–4.245, *P* = 0.025], fasting blood glucose [OR = 2.657, 95%(CI) 1.288–4.121, *P* = 0.016], glycosylated hemoglobin [OR = 3.438, 95%(CI) 2.543–4.628, *P* = 0.001], duration [OR = 1.732, 95%(CI) 1.471–3.253, *P* = 0.019] and instrument [OR = 1.472, 95%(CI) 1.112–2.288, *P* = 0.018].

**Table 3:** Univariate analysis of patient satisfaction.

Characteristics	Satisfied group (*n* = 442)	Dissatisfied group (*n* = 30)	*χ*^2^/*t* value	*P* value
Gender			0.02	0.878
Male	126	8		
Female	316	22		
Age			6.57	0.010
<70 years	176	2		
≥70 years	266	28		
Smoking			0.37	0.541
Yes	76	8		
No	290	22		
Drinking			0.44	0.510
Yes	90	4		
No	352	26		
BMI (kg/m^2^)			0.72	0.396
<22.0	101	9		
≥22.0	331	31		
BMD (T score)			0.96	0.328
<−3.0	108	10		
≥−3.0	334	20		
Fasting blood glucose			8.76	0.003
<8 mmol/L	332	18		
≥8 mmol/L	55,110	12		
Fasting C-peptide			8.44	0.004
<0.67 nmol/L	134	20		
≥0.67 nmol/L	308	10		
Glycosylated hemoglobin			10.85	0.001
<6.5 mmol/L	324	8		
≥6.5 mmol/L	118	22		
Duration			10.47	0.001
<8 years	342	24		
≥8 years	200	36		
Instrument			7.33	0.006
KMC	322	12		
Kyphon	120	18		
Fracture level			0.53	0.912
T11	102	6		
T12	110	10		
L1	130	8		
L2	100	6		

**Table 4:** Multi-factor logistic regression analysis.

Variable	Regression coefficient	Standard error	Wald value	*P*	OR	95%CI
Age	0.877	0.363	5.223	0.025	2.298	1.290–4.245
Fasting blood glucose	0.867	0.329	5.112	0.016	2.657	1.288–4.121
Fasting C-peptide	0.672	0.762	6.731	0.212	2.749	0.870–3.273
Glycosylated hemoglobin	0.837	0.526	5.802	0.001	3.438	2.543–4.628
Duration	0.502	0.987	6.543	0.019	1.732	1.471–3.253
Instrument	0.241	0.287	0.675	0.018	1.472	1.112–2.288

## Discussion

In this study, it was found in the 6-month follow-up after surgery that the overall excellent and good rate of patient satisfaction was 93.6%, indicating that the use of kyphoplasty can achieve relatively satisfactory clinical results, but there were 15 patients with poor results, and it was found that the blood glucose control of these 15 patients was not ideal, all of them were severe osteoporosis. Furthermore, during the follow-up of this study, 13 cases of compression fractures occurred in the adjacent vertebrae of surgically reshaped vertebrae and 2 cases of distal vertebral fractures. Therefore, effective control of blood glucose is an effective prerequisite for preventing fractures in diabetic patients. In addition, the results of this study showed that age ≥70 years, fasting blood glucose ≥8 mmol/L, glycosylated hemoglobin ≥6.5 mmol/L, duration ≥8 years and Kyphon instrument were independent influencing factors of OTCF complicated with T2DM.

Age is an important factor affecting the degree of osteoporosis, and bone loss increases with age (6). Previous studies have shown that one reason for bone loss with increasing age is progressive bone loss due to age-related bone remodeling imbalances with an increased bone resorption/bone formation ratio, and another is that estrogen deficiency perpetuates low levels of immune activation in a pro-inflammatory state (7, 8). Similarly, the elderly are also at high risk of diabetes (9). In addition, previous articles showed that diabetic patients had an increased risk of vertebral fractures independent of BMD or diabetic complications (10, 11). In our study, increased age was a risk factor for patient satisfaction after PKP. Zhang et al. (12) and Ning et al. (13) also showed that age was an independent risk factor for the prognosis of OTCF patients. Therefore, elderly diabetic patients should be informed that PKP may have limited efficacy.

Fasting blood glucose is one of the most frequently used factors reflecting blood glucose level in patients with T2DM. Previous studies have shown that long-term poor blood glucose control can lead to an increase in advanced glycation end products, while the continuous accumulation of advanced glycation end products can inhibit osteoblast differentiation, thus promote osteoblast apoptosis and osteoclast formation, resulting in a decrease in bone mass and aggravation of osteoporosis (14, 15). In our study, we found that fasting blood glucose ≥8 mmol/L was closely related to the satisfaction of OTCF patients with T2DM after PKP. Therefore, it is very important for diabetic patients to maintain good blood glucose control after surgery to improve the prognosis of patients. Previous articles have showed that drugs, such as metformin not only promote osteoblast proliferation, differentiation, and mineralization, but also inhibit osteoclast production and function thereby increasing bone mineral density and delaying the progression of osteoporosis (16–18). Therefore, postoperative PKP patients and T2DM can delay or prevent the progression of osteoporosis and prevent postoperative complication development by achieving good blood glucose control.

At present, most studies have confirmed that glycosylated hemoglobin ≥6.5% (≥48 mmol/mol) can irremediable long-term risk for diabetic complications and mortality (19). Lim et al. (20) analyzed 4,778 patients and showed that patients with preoperative hemoglobin A1c (HbA1c) > 8% were more likely to experience postoperative complication; indeed, in our study, glycosylated hemoglobin ≥6.5% was an independent risk factor affecting the satisfaction of OTCF patients, indicating that controlling HbA1C level <6.5% was beneficial. What's more, a systematic review showed that preoperativeHbA1c > 7.5 mg/dl was associated with an increased risk of reoperation or infection after spine surgery (21). Therefore, it is important for diabetic patients to maintain good HbA1C control to prevent the development of complications and improve patient satisfaction, especially HbA1C level more than 6.5%.

The present study demonstrated that the duration of diabetes ≥8 years was an independent risk factor for poor patient satisfaction after PKP treatment in OTCF patients with diabetes. An increasing number of studies have shown that the longer duration of diabetes affects the health-related quality of life (22, 23). Takahashi et al. (24) also showed that patients having diabetes for 20 years or more were more likely to experience poor improvement of spine surgery. Therefore, this might indicate that the surgical outcomes, perioperative complications, and patient dissatisfaction should be considered in patients with diabetes with a longer course of disease.

In our study, patients who used surgical instruments using KMC had a higher recovery rate after surgery than those who used Kyphon. Combined with the result of logistic regression analysis in this study, Kyphon instrument was independent influencing factor of OTCF complicated with T2DM. After analyzing for underlying reasons, we found some possibilities as follows: (1) When KMC was used in surgery to expand the coronal surface centered on the channel, it was not easy to produce pressure on the lateral wall of the vertebral body, so the possibility of bone cement exudating and pressing blood vessels and nerves could be minimized to the greatest extent. (2) KMC mainly expands in the height direction of the vertebral body, which can effectively restore the height of the compressed vertebral body. It is point-shaped when opened, so that the bone cement can pour into the vertebral body from all directions, while Kyphon evenly spreads the balloon, which is not easy to control in the direction and the permeability of the bone cement is not satisfactory (25, 26). The bone cement injected in KMC was more concentrated and of regular shape compared to that in Kyphon, which would concentrate greater stress on the vertebra and the load on the spine. For those who treated with PKP, they might need to choose PKM instruments to achieve better surgical results.

The limitations of the present study mainly include the following items: (1) the clinical data were collected in our hospital, so it lacked comparison with different centers. (2) The patient was followed up for a short time, so the results of long-term follow-up are needed to support our conclusions. (3) The clinical efficacy of kyphoplasty is affected by many factors, the indicators used in this study are not comprehensive, so the results are inevitably biased.

To sum up, kyphoplasty for treatment of OTCF with diabetes can play a more satisfactory clinical effect, its curative effect is affected by age, duration, fasting blood glucose levels, glycosylated hemoglobin, duration and type of instrument.

## Data Availability

The raw data supporting the conclusions of this article will be made available by the authors, without undue reservation.
